# Disruption of ruminal homeostasis by malnutrition involved in systemic ruminal microbiota-host interactions in a pregnant sheep model

**DOI:** 10.1186/s40168-020-00916-8

**Published:** 2020-09-24

**Authors:** Yanfeng Xue, Limei Lin, Fan Hu, Weiyun Zhu, Shengyong Mao

**Affiliations:** 1 Centre for Ruminant Nutrition and Feed Technology Research, College of Animal Science and Technology, Nanjing Agricultural University, Nanjing, 210095 China; 2 National Center for International Research on Animal Gut Nutrition, National Experimental Teaching Demonstration Center of Animal Science, Nanjing Agricultural University, Nanjing, 210095 China

**Keywords:** Ruminal homeostasis, Ruminal microbiota and epithelium, Energy metabolism, Signal transduction, Severe feed restriction

## Abstract

**Background:**

Undernutrition is a prevalent and spontaneous condition in animal production which always affects microbiota-host interaction in gastrointestinal tract. However, how undernutrition affects crosstalk homeostasis is largely unknown. Here, we discover how undernutrition affects microbial profiles and subsequently how microbial metabolism affects the signal transduction and tissue renewal in ruminal epithelium, clarifying the detrimental effect of undernutrition on ruminal homeostasis in a pregnant sheep model.

**Results:**

Sixteen pregnant ewes (115 days of gestation) were randomly and equally assigned to the control (CON) and severe feed restriction (SFR) groups. Ewes on SFR treatment were restricted to a 30% level of ad libitum feed intake while the controls were fed normally. After 15 days, all ewes were slaughtered to collect ruminal digesta for 16S rRNA gene and metagenomic sequencing and ruminal epithelium for transcriptome sequencing. Results showed that SFR diminished the levels of ruminal volatile fatty acids and microbial proteins and repressed the length, width, and surface area of ruminal papillae. The 16S rRNA gene analysis indicated that SFR altered the relative abundance of ruminal bacterial community, showing decreased bacteria about saccharide degradation (*Saccharofermentans* and *Ruminococcus*) and propionate genesis (*Succiniclasticum*) but increased butyrate producers (*Pseudobutyrivibrio* and *Papillibacter*)*.* Metagenome analysis displayed that genes related to amino acid metabolism, acetate genesis, and succinate-pathway propionate production were downregulated upon SFR, while genes involved in butyrate and methane genesis and acrylate-pathway propionate production were upregulated. Transcriptome and real-time PCR analysis of ruminal epithelium showed that downregulated collagen synthesis upon SFR lowered extracellular matrix-receptor interaction, inactivated JAK3-STAT2 signaling pathway, and inhibited DNA replication and cell cycle.

**Conclusions:**

Generally, undernutrition altered rumen bacterial community and function profile to decrease ruminal energy retention, promoted epithelial glucose and fatty acid catabolism to elevate energy supply, and inhibited the proliferation of ruminal epithelial cells. These findings provide the first insight into the systemic microbiota-host interactions that are involved in disrupting the ruminal homeostasis under a malnutrition pattern.

Video Abstract

## Background

In the ruminant production system, many animals are subjected to undernutrition, which may be reinforced by seasonal fluctuation of food availability or artificial control due to the economic conditions. Even if the feed supply is constant and abundant, altered nutrition requirements and intakes in specific physiological periods also induce a relatively unbalanced nutritional status. For example, the nutritional ingestion for productive animals, including pregnancy, lactation, and growth, is easily lower than the physiological demand [1]. Particularly, pregnant ewes with twins or multiple fetuses always encounter undernutrition during late gestation, since feed intake dramatically declines due to the increased volume of uterus and extruded abdomen while the nutrition requirement significantly elevates because of fetal growth and development. Previous studies revealed that undernutrition disrupted metabolic homeostasis and induced serious lipid metabolism disorders in maternal and fetal livers [2–4], which may induce maternal diseases and fetal growth retardation and maldevelopment [5–7]. However, little information is known about the effect of undernutrition on ruminal homeostasis in spite of the fact that ruminal homeostasis is the basis for the metabolism, development, and health of ruminants [8–10].

For ruminants, the rumen is a unique and vital organ. It contains highly diverse anaerobic microorganisms [11], in which bacteria are the dominant domain for microbial protein synthesis and carbohydrate digestion [12, 13]. Further, microbial proteins provide up to 90% of the amino acids reaching the small intestine [11], and volatile fatty acids (VFAs) account for more than 70% of the metabolic energy supply [14]. Besides, the ruminal epithelium is responsible for several physiologically important functions, including nutrient absorption, transport, metabolism, and barrier function. Generally, commensal microbiota can use substrates for VFA production, providing bidirectional energy sources to underpin ruminal epithelium growth. Therefore, there is a cross-metabolism pattern between microbiota and epithelium of energy supply to maintain the rumen’s natural metabolic repertoires. Previous studies focused on high-energy diets manipulating this interaction and promoting ruminal epithelium growth to adapt more VFAs [10, 15]. Consequently, the knowledge on the alternations of systemic interaction in ruminal homeostasis under malnutrition is quite limited.

Here, we hypothesized that undernutrition induced by severe feed restriction (SFR) would influence the structure and function of ruminal bacterial communities. Microbiota-derived products might regulate the critical signaling pathways in ruminal epithelium to control its metabolism and proliferation, and these effects might disrupt the rumen hemostasis. Therefore, the present study was conducted to explore the responsive mechanisms of ruminal microbiota and epithelium to undernutrition for the understanding of nutrition in maintaining ruminal homeostasis in a pregnant sheep model.

## Results

### SFR affected rumen fermentation and ruminal epithelium parameters

**Fig. 1:**
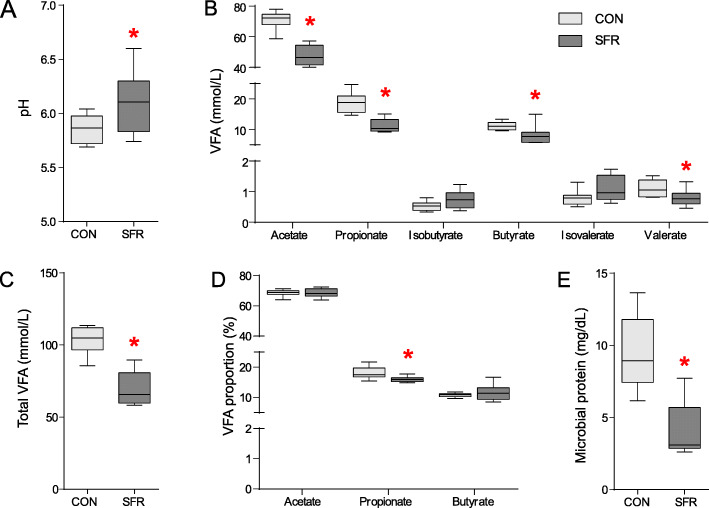
SFR changed ruminal fermentation parameters in pregnant ewes. **a** Ruminal pH. **b** The levels of ruminal VFAs. **c** Ruminal total VFA. **d** The proportions of ruminal acetate, propionate, and butyrate in total VFA. **e** Ruminal microbial protein. Data were presented as the minimum to maximum. The difference between two groups was identified by independent sample *t* test (*n* = 8 per group), and asterisk indicated the significant difference (*P* < 0.05)

Actual feed intake of the SFR and control (CON) groups was 1.50 ± 0.10 and 0.45 ± 0.03 kg/day, respectively, so the ratio of feed restriction (SFR/CON) was 29.7%. Rumen pH in the SFR group was higher (*P* = 0.040) than the CON group (Fig. 1a). As contrasted to the CON group, the concentrations of acetate (*P* < 0.001), propionate (*P* < 0.001), butyrate (*P* = 0.025), valerate (*P* = 0.039), and total VFA (*P* < 0.001) were reduced in the rumen of SFR ewes, while those of isobutyrate (*P* = 0.093) and isovalerate (*P* = 0.112) remained unchanged (Fig. 1b, c). The proportion of propionate in the SFR group was lower (*P* = 0.017) than the CON group, while those of acetate (*P* = 0.972) and butyrate (*P* = 0.402) showed no significant changes (Fig. 1d). Besides, the level of ruminal microbial protein in the SFR group was lower (*P* < 0.001) than the CON group (Fig. 1e).

**Fig. 2:**
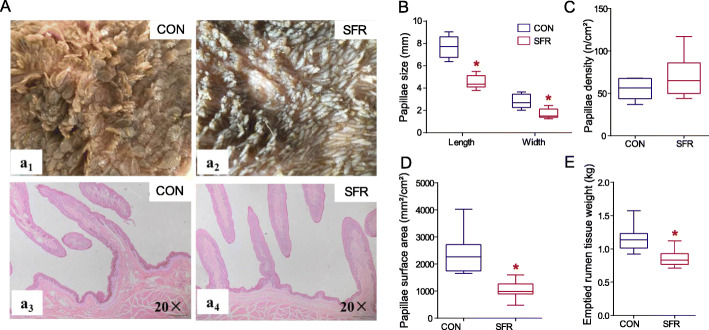
SFR affected the morphology and parameters of rumen papillae in pregnant ewes. **a** Visual inspection of rumen papillae in the CON (a_1_) and SFR (a_2_) groups. Hematoxylin and eosin staining sections of rumen papillae in the CON (a_3_) and SFR (a_4_) groups. **b** Papillae size. **c** Papillae density. **d** Papillae surface area. **e** Emptied rumen tissue weight. Data were presented as the minimum to maximum. The difference between two groups was identified by independent sample *t* test (*n* = 8 per group), and asterisk indicated the significant difference (*P* < 0.05)

Furthermore, the ruminal papillae became shorter and narrower under malnutrition (Fig. 2a). From our results, the weight of emptied rumen tissue in the SFR group was lighter (*P* = 0.003) than the CON group (Fig. 2e). SFR decreased the length (*P* < 0.001), width (*P* = 0.001), and surface area (*P* < 0.001) of papillae in the ventral sac of rumen (Fig. 2b, d). No significant difference (*P* = 0.163) was observed in the density of papillae between the two groups (Fig. 2c).

### SFR changed the structure and composition of ruminal bacteria

**Fig. 3:**
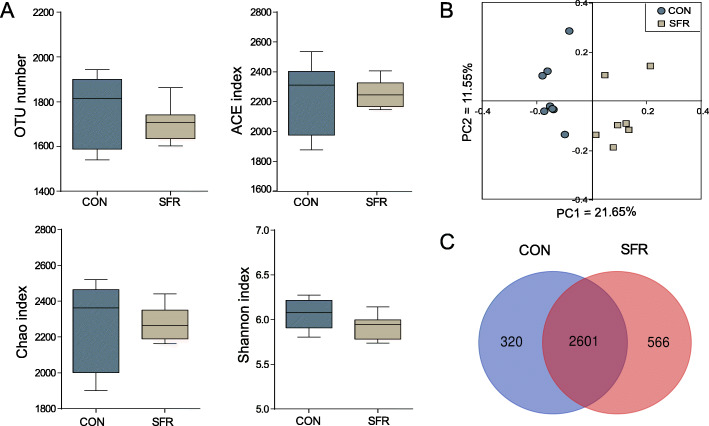
Effect of SFR on the diversity of rumen digesta-associated bacterial communities in pregnant ewes. **a** Optional taxonomic unit (OTU) number and α-diversity indexes. **b** Principal coordinate analysis of bacterial communities based on OTUs. **c** Venn diagram of OTUs in two groups. Data were presented as the minimum to maximum. The difference between two groups was identified by independent sample *t* test (*n* = 8 per group), and asterisk indicated the significant difference (*P* < 0.05)

Absolute quantification manifested total bacteria [Log_10_(copy numbers/g of rumen content)] of the SFR group (9.65 ± 0.48) was slightly lower than that of the CON group (9.79 ± 0.26), but the difference was inapparent (*P* = 0.210). To explore the mechanisms of SFR on rumen fermentation, we performed 16S rRNA gene sequencing to study the alteration of rumen bacterial communities. First, all the rarefaction curves tended to approach the plateau (Fig. S1). Then, the α-diversity showed that the OTU number (*P* = 0.374), ACE (*P* = 0.737), Chao (*P* = 0.825), and Shannon indexes (*P* = 0.083) remained unchanged (Fig. 3a). Further, both the principal coordinates analysis profile using the unweighted UniFrac metric (Fig. 3b) and a molecular variance analysis (AMOVA) (Fs = 2.500, *P* = 0.002) demonstrated the discriminant in the composition of rumen microbiota between two groups. Besides, the Venn diagram revealed that, at the OTU level, 2601 common core OTUs were shown in both groups, 320 and 566 unique OTUs distributed in the CON and SFR groups, respectively (Fig. 3c). In general, SFR changed the microbial composition in the rumen.

**Fig. 4:**
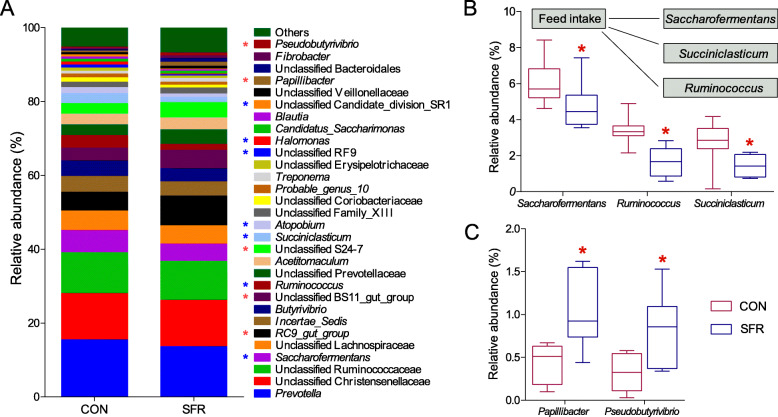
SFR changed the relative abundances of microbiota in rumen. **a** Relative abundances of bacterial communities at the genus level. Red asterisks indicated significantly increased genera while blue asterisks indicated significantly decreased genera. **b** Genera involved in carbohydrate metabolism and propionate production and their correlations with feed intake. **c** Genera involved in butyrate production. The difference between two groups was identified by non-parametric *t* test (*n* = 8 per group), and asterisk indicated the significant difference (*P* < 0.05). The Spearman correlation coefficients (*r*) and significance tests between feed intake and microbiota were calculated using bivariate correlation (*n* = 16) in SPSS 19.0, and *P* < 0.05 was used to identify significant correlations

Discriminatory characteristics were observed between the CON and SFR groups in the relative abundance of ruminal bacteria at the phylum and genus levels with the cutoff value of the average relative abundance more than 0.5% in at least one group. At the phylum level, 22 phyla were identified in both groups with Firmicutes, Bacteroidetes, and Actinobacteria being the most dominant phyla. The abundances of 5 phyla exhibited significant variability between two groups, including 2 increased phyla (BD1-5 and Elusimicrobia) and 3 reduced phyla (Actinobacteria, Tenericutes, and Candidate_division_SR1) in the SFR group (Fig. S2). At the genus level, in total, 30 taxa showed a great relative abundance (> 1% of total sequences), and 12 taxa shifted significantly between two groups (Fig. 4a). In comparison to the CON group, the relative abundances of 2 taxa involved in butyrate metabolism, including *Papillibacter* and *Pseudobutyrivibrio*, significantly increased, while the relative abundances of five taxa involved in saccharide metabolism (*Saccharofermentans* and *Ruminococcus*), propionate production (*Succiniclasticum*), and nitrogen utilization (*Atopobium* and *Halomonas*) significantly decreased in the SFR group (Fig. 4b, c). Further, the relative abundances of *Saccharofermentans*, *Ruminococcus*, and *Succiniclasticum* positively correlated with feed intake (Fig. 4b), implying their decreases resulted from substrate deficiency.

### Metabolic pathways for carbohydrate fermentation and amino acid biosynthesis by microbial cross-feeding

Metagenomic data were collected from 10 DNA samples of ruminal digesta (five from each group). Based on shotgun sequencing, we generated 254 Gb of paired-end reads, with an average of 25.4 Gb (21.2–33.1 Gb) per sample. In total, a 7.4 Gb pan-metagenome was constructed based on the assembled contigs with an average N50 length of 2.4 Kb, including 11.6 million non-redundant genes, and the average length of open read frame was 636 bp. To mechanistically probe different metabolic strategies of microorganisms under malnutrition mode, we conducted a comprehensive analysis of carbohydrate fermentation and amino acid biosynthesis.

**Fig. 5:**
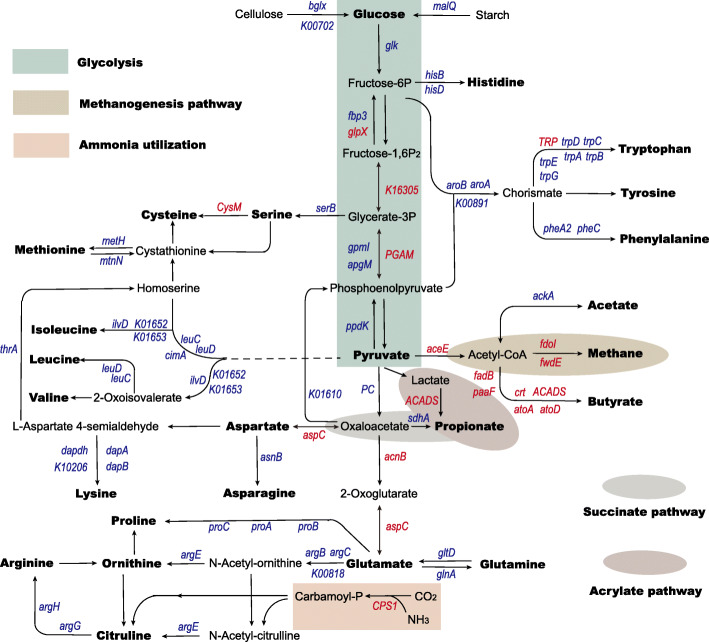
SFR changed carbohydrate metabolism and amino acid metabolism in ruminal microbiota according to metagenomic information. Comparisons of the relative abundance of KO enzymes, which related to metabolic pathways for carbohydrate fermentation and amino acid biosynthesis by microbial cross-feeding in the CON and SFR groups by the Mann−Whiney *U* test (*n* = 5 per group). Red font indicated upregulated enzyme genes while blue font indicated downregulated enzyme genes

Ruminants need efficient carbohydrate breakdown to satisfy their energy requirements, in which microbial fermentation pathways contribute to satisfying host’s needs from product fluxes. In this study, we found that genes involved in starch and cellulose degradation were downregulated in the metagenome datasets under malnutrition (Fig. 5; Table S1). For the following glucose metabolism, most genes such as *glk*/[EC:2.7.1.2], *fbp3*/[EC:3.1.3.11], *gpmI*/[EC:5.4.2.12], *apgM*/[EC:5.4.2.12], and *ppdK*/[EC:2.7.9.1] had lower abundances in SFR ewes. Subsequently, *ackA*/[EC:2.7.2.1] associated with acetate generation showed lower abundance upon SFR. For butyrate metabolism, all shifted abundances of genes, forming from the condensation of two molecules of acetyl-CoA and subsequent reduction to butyryl-CoA, were increased under the condition of SFR. For propionate generation, the genes coding enzymes in the acrylate pathway had higher abundances while those in the succinate pathway showed lower abundances in the SFR group. To better deduct microbial cross-feeding, we also paid attention to methanogenesis. The higher abundances of *fdoI*/[k00127] and *fwdE*/[EC:1.2.7.12] upon SFR implied promoted methane production. We also identified amino acid biosynthesis pathways among the high enrichment scores. The most striking discrimination was that almost all genes involved in amino acid biosynthesis showed lower abundances upon SFR (Fig. 5; Table S1).

### SFR altered the transcriptional profile in ruminal epithelium of host

Considering the substantial connections between microbiota and host as well as the huge changes of ruminal microbiota upon SFR, we performed transcriptome sequencing on ruminal epithelium samples to study the effect of microbiota on substance metabolism and signal transduction in ruminal epithelium. First, both the plots of principal component analysis and partial least squares of discriminant analysis of RNA-sequencing total genes showed a clear separation between ewes in the CON and SFR groups (Fig. S3). With the criterion of false discovery rate (FDR) < 0.05 and fold change (FC) > 1.5 or < 0.67, a total of 106 differentially expressed genes (DEGs) in ruminal epithelium were identified. To validate the transcriptome results, some DEGs were randomly selected and checked using quantitative real-time PCR (qPCR). Results of qPCR showed that the expressional levels of minichromosome maintenance (*MCM*) *2*, *MCM4*, *MCM5*, collagen (*COL*) *1A1*, *COL1A2*, *COL3A1*, and peroxisome proliferator-activated receptor (PPAR) gamma (*PPARG*) were decreased in the SFR group, while that of carnitine palmitoyl transferase (*CPT*) *1A* was increased (Fig. S4). The expressional trends of these genes were highly consistent with the transcriptome results.

**Fig. 6:**
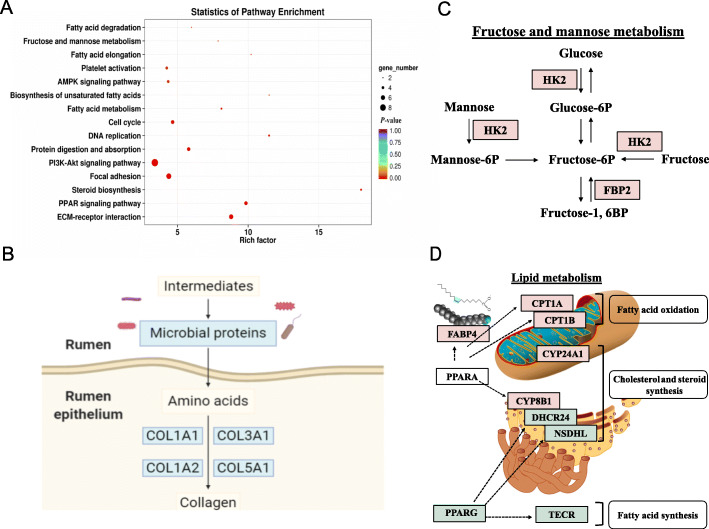
SFR changed substrate metabolism in ruminal epithelium. **a** KEGG pathway enrichment analysis of DEGs. **b** DEGs related to collagen synthesis. **c** DEGs related to fructose and mannose metabolism. **d** DEGs related to lipid metabolism. Light red, upregulated; light green, downregulated

To further analyze the DEGs, Kyoto Encyclopedia of Genes and Genomes (KEGG) pathway enrichment analysis was conducted. As shown in Fig. 6a, the enriched top 15 pathways included extracellular matrix (ECM)-receptor interaction, PPAR signaling pathway, steroid biosynthesis, focal adhesion, phosphoinositide 3 kinase-protein kinase B (PI3K-AKT) signaling pathway, protein digestion and absorption, cell cycle, DNA replication, fatty acid metabolism, biosynthesis of unsaturated fatty acids, AMPK signaling pathway, fatty acid elongation, fructose and mannose metabolism, and fatty acid degradation.

### SFR changed substrate metabolism in ruminal epithelium

Collagen, the main structural protein in extracellular space, is the richest protein in mammals, which is coded by the collagen gene family. In ruminal epithelia of SFR ewes, downregulated *COL1A1*, *COL1A2*, *COL3A1*, and *COL5A1* suggested low ability of collagen synthesis (Fig. 6b). This could be explained by the declined microbial protein production from microbiota, which might affect signal transduction between extracellular factors and intracellular response. Regarding carbohydrate metabolism, upregulated hexokinase 2 (*HK2*) and fructose-1,6-bisphosphatase 2 (*FBP2*) in the ruminal epithelia of SFR ewes suggested enhanced fructose and mannose metabolism to increase energy production (Fig. 6c).

The most interesting thing was that many enriched pathways were involved in lipid metabolism and PPAR signaling. Genes regulated by PPAR alpha (*PPARA*) signaling pathway, including *FABP4* (participating in fatty acids uptake and intracellular transport), *CPT1A* and *CPT1B* (controlling mitochondrial fatty acid oxidation), and cytochrome P450 (*CYP*) *8A1* (linking to cholesterol and steroid synthesis in the endoplasmic reticulum), were upregulated in the ruminal epithelia of SFR ewes. Genes regulated by PPARG signaling pathway, including 24-dehydrocholesterol reductase (*DHCR24*) and NAD(P)-dependent steroid dehydrogenase-like (*NSDHL*) (referring to cholesterol and steroid synthesis in the endoplasmic reticulum) and trans-2,3-enoyl-CoA reductase (*TECR*) (relating to fatty acid biosynthesis and elongation in the cytoplasm), were downregulated along with the decreased expression of *PPARG* in the ruminal epithelia of SFR ewes (Fig. 6d). Taken together, SFR enhanced saccharide metabolism and fatty acid oxidation to increase energy supply and repressed fatty acid synthesis to decrease energy expenditure.

### SFR downregulated JAK3-STAT2 signaling pathway and inhibited ruminal epithelial cell proliferation

**Fig. 7:**
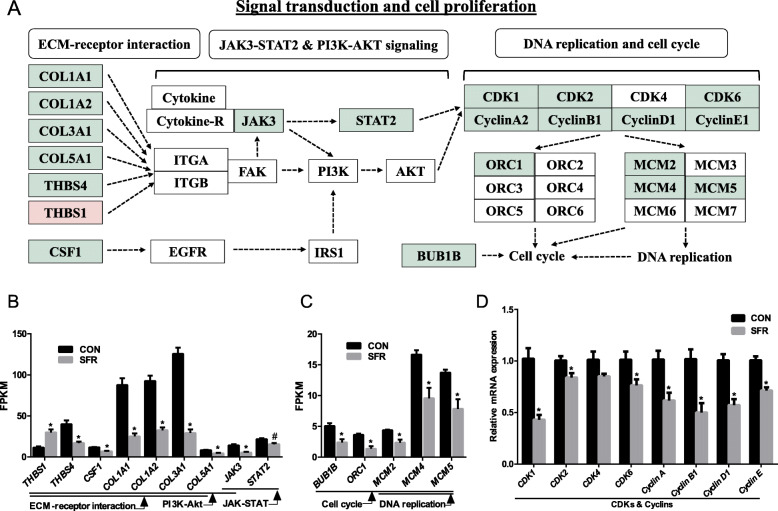
SFR inactivated signal transduction in the ruminal epithelial cells and inhibited cell proliferation. **a** Schematic plot of signal transduction involved in JAK3-STAT2 and PI3K-AKT signaling pathways. Light red, upregulated; light green, downregulated. **b** DEGs related to JAK3-STAT2 and PI3K-AKT signaling pathway in RNA sequencing. **c** DEGs related to DNA replication and cell cycle in RNA sequencing. **d** Genes related to cyclins and cyclin-dependent kinases in quantitative real-time PCR. Data were presented as the mean with SEM. DEGs were selected based on FDR < 0.05 and FC > 1.5 or < 0.67 (*n* = 5 per group). Pound key of STAT2 indicated that *P* < 0.05 and FDR > 0.05. The difference of gene expression in quantitative real-time PCR was identified by independent sample *t* test (*n* = 8 per group), and asterisk indicated the significant difference (*P* < 0.05)

Both cell cycle and DNA replication were enriched by DEGs in KEGG pathway analysis (Fig. 6a). Further, DEGs enriched in DNA replication including *MCM2*, *MCM4*, and *MCM5* and cell cycle including *BUB1B*, *ORC1*, *MCM2*, *MCM4*, and *MCM5* were all downregulated in the ruminal epithelia of SFR ewes (Fig. 7a, c). Considering these genes were regulated by cyclins and cyclin-dependent kinases (CDKs), we probed the expressional levels of *CDK1*, *CDK2*, *CDK4*, *CDK6*, *cyclinA2*, *cyclinB1*, *cyclinD1*, and *cyclinE1* using qPCR. As expected, the expressional levels of *CDK1* (*P* < 0.001), *CDK2* (*P* = 0.028), *CDK6* (*P* = 0.035), *cyclinA2* (*P* = 0.009), *cyclinB1* (*P* = 0.004), *cyclinD1* (*P* < 0.001), and *cyclinE1* (*P* < 0.001) were decreased under the condition of SFR (Fig. 7a, d). PI3K-AKT signaling pathway plays an important role in regulating CDKs and cyclins and involves in cell proliferation [16], which was significantly enriched in KEGG pathway analysis. However, the expression of PI3K and AKT themselves remained unchanged upon SFR. Interestingly, Janus kinase 3 (JAK3), a member in PI3K-AKT signaling pathway, was downregulated in the SFR group. Further, its down-stream transcriptional factor signal transducer and activator of transcription 2 (STAT2) was also downregulated (*P* = 0.019) upon SFR even though it was not identified as a DEG (FDR > 0.05) (Fig. 7a, b). JAK-STAT signaling pathway plays a critical role in cell cycle progression and anti-apoptosis [17] and it is highly correlated with ECM-receptor interaction which senses extracellular factors and regulates intracellular signaling transduction. For ECM-receptor interaction, collagens can bind with membrane receptors integrin alpha (ITGA) and beta (ITGB) to induce focal adhesion kinase (FAK) activation [18]. Thrombospondin (THBS) 1 and THBS4 are two members of THBS family—multidomain matrix glycoproteins—which can interact with cell adhesion receptors to positively and negatively modulate the adhesion, motility, and growth of epithelial cells [19]. Colony-stimulating factor 1 (CSF1) binds with epidermal growth factor receptor (EGFR) and acts on insulin receptor substrate 1(IRS1). RNA-sequencing data showed that *COL1A1*, *COL1A2*, *COL3A1*, *COL5A1*, *THBS4*, and *CSF1* were all downregulated in the ruminal epithelia of SFR ewes while only *THBS1* was upregulated (Fig. 7a, b). Taken together, our results hint that SFR inhibited ECM-receptor interaction and then repressed JAK3-STAT2 signaling transduction, which subsequently downregulated the expressional levels of CDKs and cyclins in cell cycle.

## Discussion

In the current study, we deeply dissected the effect of undernutrition on microbial composition and metabolism and then the cascades of signal transduction and tissue renewal in ruminal epithelium, contributing to clarifying the detrimental effect of undernutrition on ruminal homeostasis in a pregnant sheep model. Our results indicated that SFR decreased the concentrations of ruminal acetate, propionate, butyrate, valerate, and total VFA, which is similar to the results in cattle under fasting or decreased feed intake [20, 21]. This could be explained by the lack of available fermentable substrates and the alteration of ruminal microbial communities in SFR group. Among these changed genera mentioned earlier, *Saccharofermentans* can ferment hexoses, polysaccharides, alcohols, sucrose, and aesculin to produce acetate, lactate, and fumarate [22]; *Succiniclasticum* converts succinate to propionate, which is an important energy-yielding mechanism in rumen [23]; *Ruminococcus* may participate in breaking down the plant cell wall [24]. Thus, the lower abundances of *Saccharofermentans*, *Succiniclasticum*, and *Ruminococcus* upon SFR, which were highly possibly caused by decreased fermentable substrates, implied the declined ability of ruminal saccharide metabolism. *Pseudobutyrivibrio* can ferment a variety of carbohydrates with butyrate as an important end product [25]. *Papillibacter* is also a known butyrate producer [26]. Thus, the increased abundances of *Pseudobutyrivibrio* and *Papillibacter* upon SFR suggested the proportion of butyrate production by rumen microbiota was relatively increased. However, even so, this could not rescue the decreased level of butyrate due to low fermentative substrates but just kept the unchanged percentage of butyrate. In addition, *Atopobium* produces ammonia in rumen [27], which was found to be increased in cattle’s rumen upon high-grain diet [28]. Some *Halomonas* species can gain energy through denitrification by converting nitrate to nitrogen [29]. The lower abundances of *Atopobium* and *Halomonas* upon SFR may suggest the low efficiency of nitrogen conversion and utilization.

To explore whether malnutrition-induced alterations of microbial communities caused microbial functional differences, we used metagenome sequencing to analyze the entire metabolic pathways. Metagenomic results showed that the pathways involved in microbial carbohydrate fermentation and amino acid biosynthesis were heavily influenced by malnutrition. Underwent the decrease of feed intake, downregulated conversion of cellulose and starch to glucose and biodegradation of glucose to pyruvate implied that the reduced fermentation precursors might be a driving force for lower fermentation products (VFAs) [10]. We continued to seek pyruvate metabolism, including acetate, propionate, and butyrate biosynthesis and methanogenesis. We discovered that microorganisms concentrated more energy on butyrate production than acetate production upon SFR, which was also supported by the increased abundances of butyrate-producing bacteria *Pseudobutyrivibrio* and *Papillibacter.* Propionate is mainly produced via the succinate pathway (from pyruvate to succinate and then to propionate) and the acrylate pathway (using acrylate and lactate as substrates) [30, 31]. We found that propionate production through the acrylate pathway was probably increased while that through the succinate pathway was weakened under SFR condition. Additionally, the enhanced methanogenesis in SFR ewes was in agreement with the report by Goopy et al. [32] who found that severe below-maintenance feed intake increased methane yield in cattle. As well-known that methanogenesis competes the same substrates such as hydrogen with propionate production [31, 33]. Thus, the enhanced methanogenesis suggests that malnutrition may exclude propionate-producing functional groups [34–36]. These finding also revealed that malnutrition resulted in a decrease in energy retention in rumen. Following carbohydrate fermentation, we also identified amino acid biosynthesis pathways using carbohydrate metabolism products as precursors for multilayered reactions. An interesting discovery was that amino acid biosynthesis was extremely repressed upon SFR which was also underpinned by the decreased microbial protein content. This could be likely related to the decreased abundance of genes involved in precursor synthesis and the reduction of these precursors themselves, including fructose-6P, glycerate-3P, phosphoenolpyruvate, pyruvate, and oxaloacetate [37]. Besides, the decreased ruminal microbial protein synthesis might also link to both the lower VFA production and nitrogen availability as mentioned earlier. Taken together, these findings showed that malnutrition disrupted ruminal homeostasis to drive rumen microbial function shift through different subsets, making microbes a link between diet and different physiological states.

Considering the close ties between microbiota and host, the lower ability of energy production and protein synthesis by rumen microbiota upon SFR might influence ruminal epithelium metabolism. In line with our speculation, KEGG pathway enrichment of DEGs demonstrated the metabolic changes of proteins, carbohydrates, and lipids. Among these metabolic alternations, downregulated protein synthesis in the ruminal epithelia of SFR ewes was possibly caused by the decreased amino acid metabolism in ruminal microbiota because microbial protein is the major sources of amino acids in ruminants. VFAs generated by ruminal microbial fermentation are known to be the main energy source for ruminants, so the lower concentrations of VFAs indicated the shortage of available energy in SFR ewes. Upregulated genes linked to carbohydrate metabolism in the ruminal epithelium implied the host enhanced saccharide conversion and made efforts to alleviate the shortage of energy. However, even so, it could not offset the general energy deficiency. Therefore, fatty acid oxidation regulated by PPARA was enhanced to elevate energy supply. At the same time, fatty acid synthesis and cholesterol and steroid synthesis regulated by PPARG were inhibited to reduce energy expenditure in ruminal epithelium in SFR ewes. Generally, these findings revealed that decreased energy production and microbial protein synthesis by ruminal microbiota changed the metabolic flux of proteins, carbohydrates, and lipids in ruminal epithelium to enhance energy production and diminish energy expenditure upon SFR.

Ruminal epithelial tissue plays a key role in VFA absorption, and this ability is highly dependent on the number and size of ruminal papillae. Previous studies revealed that the adaptation of ruminal epithelium to highly fermentable diets entailed morphological adaptations associated with tissue proliferation [9, 10], indicating the ruminal morphology are important in maintaining the ruminal homeostasis. Our data revealed that undernutrition decreased the empty rumen tissue weight and the length, width, and surface area of rumen papillae, suggesting that malnutrition disrupted morphological homeostasis in ruminal epithelium. We also explored the mechanism of signal transduction in ruminal epithelium upon malnutrition. ECM-receptor interaction mainly controls cell adhesion, migration, proliferation, and coagulation cascade activation [38], downregulation of genes linked to this pathway demonstrated that SFR weakened the interactions between ECM and membrane receptors and barriered the activation of intracellular signaling pathway. JAK-STAT signaling pathway has been reported as a vital intracellular mediator implicated in various functions such as survival, proliferation, differentiation, and anti-apoptosis [17]. Our results indicated that the blunted JAK3-STAT2 signaling pathway in ruminal epithelium upon undernutrition might fail to facilitate CDKs transcription. Subsequently, both MCM complex and origin recognition complex (ORC) are highly conserved 6-subunit complexes that relate to initiate genome replication in eukaryotes. The hexameric MCMs complex, which is phosphorylated and regulated by CDKs [39], participates in replication fork formation and recruits other proteins for DNA replication [40]. ORC specifically binds to the origins of replication and serves the assembly of pre-replication complex as a platform [41, 42]. ORC1 is the largest subunit of ORC, and its protein level varies during cell cycle [43]. *BUB1B* plays an important role in spindle checkpoint function and chromosome separation [44]. In the current study, downregulated *MCM2*, *MCM4*, *MCM5*, *ORC1*, and *BUB1B* in the ruminal epithelia of SFR ewes confirmed that DNA replication and cell cycle were repressed to a great extent (Fig. 7a, c). DNA replication inhibition and cell cycle arrest were bound to affect the proliferation and update of ruminal epithelial cells; finally, this changed the morphology of ruminal papillae to be much shorter and narrower.

## Conclusions

**Fig. 8:**
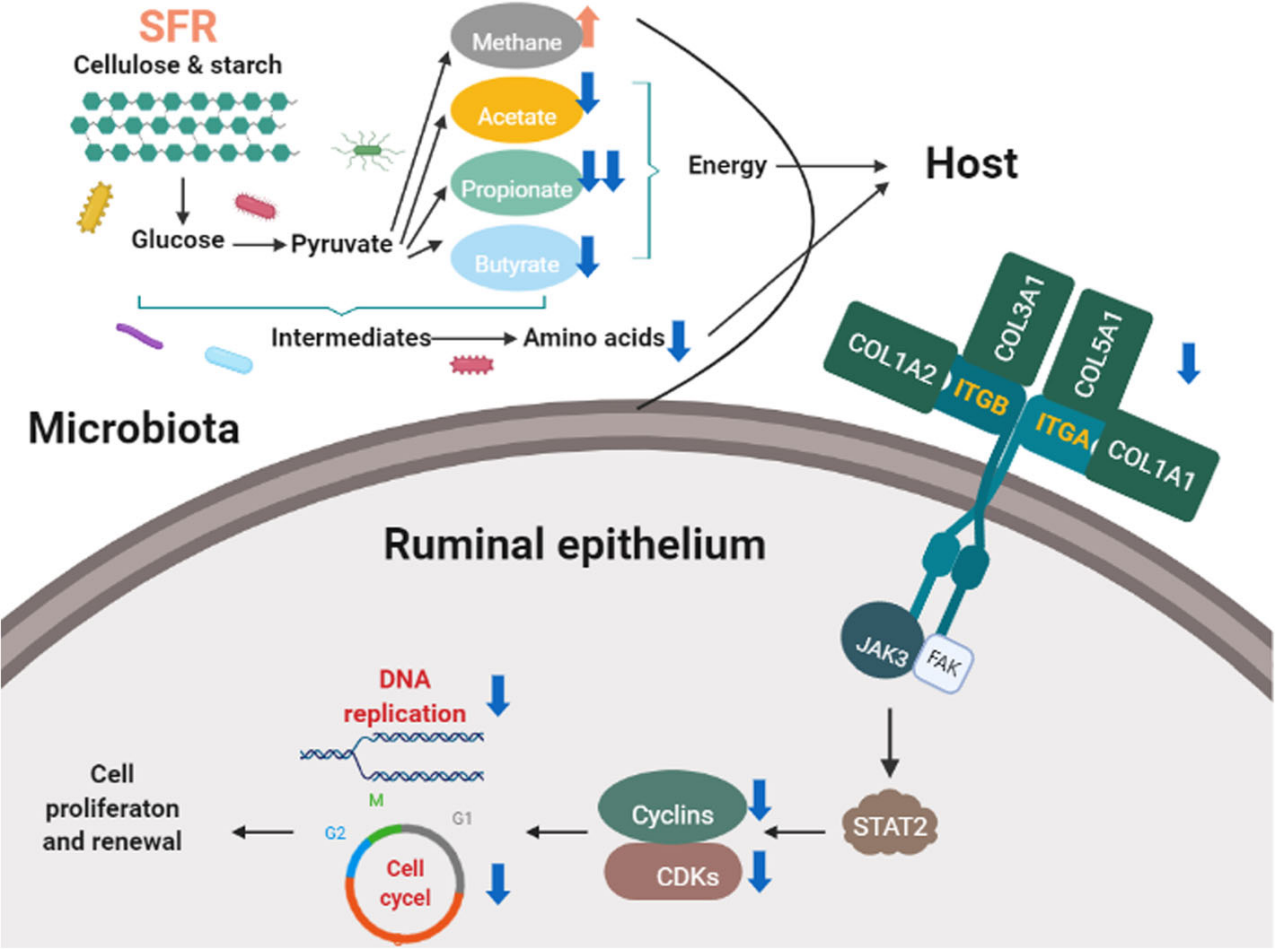
Comprehensive response of ruminal microbiota and epithelium to undernutrition and the crosstalk between them

In summary, our study revealed that SFR decreased the concentrations of VFAs and especially propionate proportion and altered the composition of ruminal bacterial communities. Furthermore, SFR decreased the intermediates of carbohydrate metabolism and repressed amino acid synthesis in ruminal microbiota. Less energy and microbial proteins could be provided to host by ruminal microbiota, which depressed the ECM-receptor interaction in ruminal epithelium and inactivated intracellular JAK3-STAT2 signaling pathway. This inhibited the expression of cyclins and CDKs and subsequently downregulated MCM complex and ORC. Finally, DNA replication inhibition and cell cycle arrest repressed the proliferation and renewing of epithelial cells, resulting in the morphologic changes of rumen papillae (Fig. 8). These findings provide new insights into the systemic microbiota-host interactions in disrupting the ruminal homeostasis under malnutrition pattern. It will be helpful in further developing nutritional regulation strategies to attenuate energy shortage during late gestation in ruminants.

## Methods

### Animal, diets, and experimental design

This study was a part of a larger project which aimed to explore how undernutrition during late gestation affected maternal and fetal metabolic homeostasis. Animal feeding and management was described previously by Xue et al. [45]. Briefly, 20 ewes (body weight 60.6 ± 4.9 kg, 2–3 parity, and pregnant for 108 days) were fed ad libitum to evaluate feed intake baseline in the 7-day adaptive period, then pregnant ewes were assigned randomly to the CON group (*n* = 10, fed with the feed intake baseline) and SFR group (*n* = 10, restricted to 30% level of the feed intake baseline) for 15 days. Ewes were fed twice a day (09:00 and 15:00) with free access to water. The diet was total mixed ration, which contained concentrate (maize, soybean meal, barley, and premix), oat hay, and rye silage. The digestible energy and crude protein content in the diet were 14.78 MJ/kg and 14.71%, respectively. The detailed ingredient compositions and nutrition levels are presented in Table S2. Ewes were slaughtered 4 h after morning feeding, and we collected ruminal digesta and epithelium samples from 16 ewes (8 ewes in each group). Power calculation had identified a required sample size of 8 ewes per group to enable detection of an effect size of 1.94 for most of the cognitive test scores with 95% power and a type I error of 5%.

### Rumen pH and fermentation parameter determination

A part of the ruminal digesta sample was immediately stored in a − 20 °C freezer for microbial DNA extraction, while another part was promptly strained through 4-layer gauze to obtain rumen fluid for pH determination. Thereafter, 25% (wt/vol) metaphosphoric acid was added in the rumen fluid and preserved at − 20 °C for later measurement of ruminal VFAs. The concentrations of VFAs were determined by gas chromatography (GC-14B, Shimadzu, Japan; capillary column film thickness: 30 m × 0.32 mm × 0.25 μm; column temperature 130 °C; injector temperature 180 °C; detector temperature 180 °C) [46]. The Coomassie Brilliant Blue G-250 assay was employed to determine the microbial protein levels in the rumen [47].

Meanwhile, tissues from the rumen ventral sac were collected and rinsed 3 times in ice-cold PBS to remove feed particles. The collected ruminal tissues were divided into 3 portions. For the first part, ruminal epithelium was separated from the muscular and serosal layers by blunt dissection and stored in liquid nitrogen for RNA extraction. The second part was stored in 4% paraformaldehyde to make hematoxylin and eosin staining sections for morphologic observation. For the third part, the papillae were cut from the ventral rumen tissue (1 cm × 1 cm) to determine the length, width, and density of papillae through the method described by Malhi et al. [48]. The papillae surface area (mm^2^/cm^2^) was calculated as the length × width × density × 2.

### Rumen bacterial DNA isolation, amplification, sequencing, and analysis

We used the bead beating method to break the cell structures of microorganisms in 0.3 g ruminal digesta and extracted DNA according to the CTAB method [49]. Then, the quality and concentration of each DNA sample was measured on the Nanodrop spectrophotometer (Thermo, Madison, Wisconsin, USA). QuantStudio 5 Real-time PCR Instrument (Applied Biosystems, Foster, California, USA) was used to analyze the absolute abundance of total bacteria according to the method, including reaction mixtures and PCR programs, described by Konstantinov et al*.* [50]. The forward and reverse primers targeting total bacteria were (5′-GTGSTGCAYGGYYGTCGTCA-3′) and (5′-ACGTCRTCCMCNCCTTCCTC-3′), respectively [51]. The bacterial 16S rRNA genes primers were 338F (5′-barcode- ACTCCTRCGGGAGGCAGCAG-3′) and 806R (5′-GGACTACCVGGGTAT CTAAT-3′) [52]. The V3-V4 region was amplified by the PCR reaction in the 20 μL mixture. Amplicons were purified using the AxyPrep DNA Gel Extraction Kit (Axygen Biosciences, Union City, CA, USA); after that, the PCR production was used to construct a sequencing bank with the Illumina TruSeq DNA Sample Preparation Kit (Illumina, San Diego, CA, USA). Illumina TruSeq PE Cluster and Sequencing by Synthesis Kits were applied to perform cluster generation, template hybridization, isothermal amplification, linearization, blocking and denaturation, and hybridization of the sequencing primers. Paired-end sequencing 2 × 250 bp was performed to sequence all libraries on an Illumina MiSeq platform according to standard protocols. Raw FASTQ data was processed by the QIIME (version 1.9.0) [53]. Then, OTUs were clustered with the 97% similarity level using UPARSE (version 7.1) [54], and chimeric sequences were identified using UCHIME [55]. The most prevalent sequences within each OTU aligned with the SILVA database and were designed as representative sequences [56]. Rarefaction curves were carried out to evaluate the sequencing depth. The α diversity was performed to estimate the bacterial diversity. The unweighted UniFrac distance metrics and AMOVA were carried out to assess the significant difference among the 16 samples [57].

### Shotgun metagenome sequencing and analysis

The method of shotgun metagenome sequencing and analysis referred to the description of Lin et al. [10]. Ten DNA samples of rumen microbiota (five from each group) were randomly selected to construct metagenomic DNA libraries using Illumina’s Truseq. Libraries were pooled and conducted paired-end sequencing on an Illumina HiSeq PE 150 Platform. Subsequently, FastQC (version 0.11.8) [58] and BWA (version 0.7.12) [59] were utilized to remove the adaptors, low-quality reads, and *ovis aries* contaminations in sequencing raw data. Then, MEGAHIT (version 1.1.1) [60] was used to assemble the obtained clean reads based on the option of min-contig-len 500, and Salmon [61] was used to exclude the contigs whose coverages were not higher than 60%. We used Prodigal (version 2.6.3) [62] to do rumen microbiota gene prediction based on contigs from each sample and took advantage of CD-HIT [63] to cluster assembled contigs based on 95% cutoff sequencing identity. Finally, we utilized the pan-metagenome to analyze the alteration of metagenome functions in ewes’ rumen upon feed restriction during late gestation.

### Transcriptome analysis of ruminal epithelium samples

Trizol method described by Chomcyznski and Sacchi [64] was used to extract total RNA from ruminal epithelium. The RNA concentration was then quantified using a Nanodrop spectrophotometer ND-1000 (Thermo Fisher Scientific, Madison, WI). The absorption ratio (260/280 nm) of all samples was between 1.8 and 2.1, indicating high RNA purity. The Agilent Bioanalyzer 2100 system (Agilent Technologies, CA, USA) with RNA Nano 6000 Assay Kit was used to check the RNA integrity to make sure integrity number was between 8 and 10 and the ratio of 28S/18S ranged from 1.8 to 2.0. Thereafter, 10 total RNA samples (five from each group) were selected randomly for cDNA library preparation.

The poly-T oligo-attached magnetic beads were used to isolate mRNA from total RNA, which was subsequently fragmented (the average length was about 200 bps) and converted to cDNA by reverse transcription. The cDNA was purified using the Qubit® dsDNA HS Assay Kit and then attached with sequencing adaptors. Fragments with suitable length (approximately 200–300 bps) were isolated using the NEBNext® Ultra™ RNA Library Prep Kit and amplified by PCR. The quality of cDNA libraries was checked by Agilent Bioanalyzer 2100 system. At last, the libraries were paired-end sequenced at the Biomarker company (Beijing, China) using Illumina Hi-Seq 2500 platform.

Clean reads were generated by removing low-quality reads, reads with adaptor sequences, and reads with more than 5% unknown bases in raw reads, which were then aligned to the *ovis aries* reference genome 3.1 using Top-Hat 2.0.9 [65]. The fragments per kilobase of transcript per million fragments mapped values were calculated to demonstrate the expression levels of genes. The DEGs were selected by the threshold values: FC > 1.5 or < 0.67 and FDR < 0.05. Finally, the major public pathway-related database KEGG was used to conduct pathway enrichment analysis of DEGs by the KOBAS 2.0 software [66].

### qPCR analysis of genes in ruminal epithelium

Total RNA was used for reverse transcription using a PrimeScript® RT reagent kit with gDNA Eraser (Takara Bio, Otsu, Japan). The expression of target genes was determined on the QuantStudio 5 Real-time PCR Instrument (Applied Biosystems, Foster, California, USA) with fluorescence detection of SYBR green dye under the standard program [45]. The data of the gene expression were normalized by the housekeeping gene (glyceraldehyde 3-phosphate dehydrogenase, *GAPDH*) using the 2^−∆∆CT^ method [67]. The primers and amplicon sizes of genes are shown in Table S3.

### Statistical analysis

The independent sample *t* test in SPSS 19.0 (SPSS Inc., Chicago, IL, USA) was performed to assess the differences of feed intake, rumen fermentation parameters, rumen papillae size, absolute abundance of total bacteria, and the mRNA expressional levels of genes in ruminal epithelium between the CON and SFR groups. The Mann-Whitney *U* test in SPSS 19.0 was used to evaluate the differences of the abundances of bacterial communities and metagenomic enzyme coding genes between two groups. The value of *P* < 0.05 was statistically significant. The Spearman correlation coefficients (*r*) and significance tests between feed intake and microbiota were calculated using bivariate correlation (*n* = 16) in SPSS 19.0, and *P* < 0.05 was used to identify significant correlations.

## Supplementary information

**Additional file 1:.** Supplementary Fig. S1 Rarefaction curves based on operational taxonomic units (OTUs) at 3% divergence for each rumen epithelium.

**Additional file 2: **Supplementary Fig. S2 SFR changed the relative abundances of microbiota at phylum level in rumen. The difference between two groups was identified by non-parametric t-test (n = 8 per group), and asterisk indicated the significant difference (*P* < 0.05).

**Additional file 3:.** Supplementary Table S1 Effect of SFR on the relative abundance of enzyme genes related to carbohydrate and amino acid metabolism based on metagenome analysis.

**Additional file 4:.** Supplementary Fig. S3 The PCA and PLS-DA of total genes in the ruminal epithelium of the CON and SFR groups. (a) The PCA score scatter plot; (b) The PLS-DA score scatter plot [predictive ability parameter (Q^2^) (cum) = 0.594, goodness-of-fit parameter (R^2^) (Y) = 0.980]. PCA, principal components analysis; PLS-DA, partial least squares of discriminant analysis.

**Additional file 5: **Supplementary Fig. S4 Validation of RNA-sequencing data using quantitative real-time PCR. Data were represented as the mean with SEM. The difference of gene expression was identified by independent-sample t-test (n = 8 per group), and asterisk indicated the significant difference (*P* < 0.05).

**Additional file 6:.** Supplementary Table S2 Ingredient composition and nutritional level of the total mixed ration.

**Additional file 7:.** Supplementary Table S3 Gene primers used for quantitative real time-PCR.

## Data Availability

Raw reads of 16 s rRNA gene sequencing of ruminal microbiota are available at National Center for Biotechnology Information (NCBI) Sequence Read Archive (SRA) (project number SRP176428 and accession number PRJNA513129). Raw reads of metagenomic sequencing of ruminal epithelium are available at NCBI SRA (project number PRJNA601318 and accession number SRP242661). Raw reads of transcriptome sequencing of ruminal epithelium are available at NCBI SRA (project number PRJNA513119 and accession number SRP175475).

## Abbreviations

AMOVA: Molecular variance analysis
CON: Control
DEGs: Differentially expressed genes
ECM: Extracellular matrix
FC: Fold change
FDR: False discovery rate
GO: Gene ontology
JAK-STAT: Janus kinase-signal transducer and activator of transcription
KEGG: Kyoto Encyclopedia of Genes and Genomes
OTU: Operational taxonomic unit
PI3K-AKT: Phosphoinositide 3 kinase-protein kinase B
PPAR: Peroxisome proliferator-activated receptor
qPCR: Quantitative real-time PCR
SFR: Sever feed restriction
VFA: Volatile fatty
