# Supplementary material for: Disruption of ruminal homeostasis by malnutrition involved in systemic ruminal microbiota-host interactions in a pregnant sheep model
Source: Microbiome. 2020 Sep 24;8:138. doi: 10.1186/s40168-020-00916-8 (PMC7517653; doi:10.1186/s40168-020-00916-8)
Supplement: Supplementary file 2 — Additional file 1:. Supplementary Fig. S1 Rarefaction curves based on operational taxonomic units (OTUs) at 3% divergence for each rumen epithelium. [file 40168_2020_916_MOESM1_ESM.docx]

**Additional file 1**

**Supplementary Fig. S1** Rarefaction curves based on operational taxonomic units (OTUs) at 3% divergence for each rumen epithelium.
