# Supplementary material for: Disruption of ruminal homeostasis by malnutrition involved in systemic ruminal microbiota-host interactions in a pregnant sheep model
Source: Microbiome. 2020 Sep 24;8:138. doi: 10.1186/s40168-020-00916-8 (PMC7517653; doi:10.1186/s40168-020-00916-8)
Supplement: Supplementary file 3 — Additional file 2: Supplementary Fig. S2 SFR changed the relative abundances of microbiota at phylum level in rumen. The difference between two groups was identified by non-parametric t-test (n = 8 per group), and asterisk indicated the significant difference (P < 0.05). [file 40168_2020_916_MOESM2_ESM.docx]

**Additional file 2**

**Supplementary Fig. S2** SFR changed the relative abundances of microbiota at phylum level in rumen. The difference between two groups was identified by non-parametric t-test (n= 8 per group), and asterisk indicated the significant difference (*P* < 0.05).
