# Supplementary material for: Disruption of ruminal homeostasis by malnutrition involved in systemic ruminal microbiota-host interactions in a pregnant sheep model
Source: Microbiome. 2020 Sep 24;8:138. doi: 10.1186/s40168-020-00916-8 (PMC7517653; doi:10.1186/s40168-020-00916-8)
Supplement: Supplementary file 4 — Additional file 3:. Supplementary Table S1 Effect of SFR on the relative abundance of enzyme genes related to carbohydrate and amino acid metabolism based on metagenome analysis. [file 40168_2020_916_MOESM3_ESM.docx]

**Additional file 3**

**Supplementary Table S1** Effect of SFR on the relative abundance of enzyme genes related to carbohydrate and amino acid metabolism based on metagenome analysis

| Entry | Gene symbol | Gene name | *P-*value | Change |
| --- | --- | --- | --- | --- |
| K00702 | *cp* | cellobiose phosphorylase | 0.016 | Down |
| K00705 | *malQ* | 4-alpha-glucanotransferase | 0.008 | Down |
| K05349 | *bglX* | beta-glucosidase | 0.008 | Down |
| K00239 | *sdhA* | succinate dehydrogenase | 0.008 | Down |
| K00845 | *glk* | glucokinase | 0.008 | Down |
| K00925 | *ackA* | acetate kinase | 0.008 | Down |
| K01006 | *ppdK* | pyruvate orthophosphate dikinase | 0.016 | Down |
| K01610 | *pckA* | phosphoenolpyruvate carboxykinase | 0.008 | Down |
| K01958 | *PC* | pyruvate carboxylase | 0.032 | Down |
| K04041 | *fbp3* | fructose-1,6-bisphosphatase III | 0.008 | Down |
| K15633 | *gpm* | 2,3-bisphosphoglycerate-independent phosphoglycerate mutase | 0.008 | Down |
| K15635 | *apgM* | imidazoleglycerol-phosphate dehydratase / histidinol-phosphatase | 0.032 | Down |
| K01089 | *hisB* | histidinol-phosphatase | 0.032 | Down |
| K00013 | *hisD* | histidinol dehydrogenase | 0.016 | Down |
| K01735 | *aroB* | 3-dehydroquinate synthase | 0.008 | Down |
| K00891 | *aroK* | shikimate kinase | 0.008 | Down |
| K00800 | *aroA* | 3-phosphoshikimate 1-carboxyvinyltransferase | 0.016 | Down |
| K01657 | *trpE* | anthranilate synthase component I | 0.032 | Down |
| K01658 | *trpG* | anthranilate synthase component II | 0.032 | Down |
| K00766 | *trpD* | anthranilate phosphoribosyltransferase | 0.008 | Down |
| K01609 | *trpC* | indole-3-glycerol phosphate synthase | 0.008 | Down |
| K01695 | *trpA* | tryptophan synthase alpha chain | 0.008 | Down |
| K01696 | *trpB* | tryptophan synthase beta chain | 0.032 | Down |
| K04518 | *pheA2* | prephenate dehydratase | 0.008 | Down |
| K01713 | *pheC* | cyclohexadienyl dehydratase | 0.008 | Down |
| K01079 | *serB* | phosphoserine phosphatase | 0.016 | Down |
| K01243 | *mtnN* | adenosylhomocysteine nucleosidase | 0.008 | Down |
| K00548 | *metH* | 5-methyltetrahydrofolate--homocysteine methyltransferase | 0.008 | Down |
| K12524 | *thrA* | bifunctional aspartokinase/homoserine dehydrogenase 1 | 0.008 | Down |
| K01953 | *asnB* | asparagine synthase | 0.016 | Down |
| K01714 | *dapA* | 4-hydroxy-tetrahydrodipicolinate synthase | 0.032 | Down |
| K00215 | *dapB* | 4-hydroxy-tetrahydrodipicolinate reductase | 0.008 | Down |
| K10206 | *dapat* | diaminopimelate aminotransferase | 0.032 | Down |
| K03340 | *dapdh* | diaminopimelate dehydrogenase | 0.008 | Down |
| K01703 | *leuC* | 3-isopropylmalate/(R)-2-methylmalate dehydratase large subunit | 0.032 | Down |
| K01704 | *leuD* | 3-isopropylmalate/(R)-2-methylmalate dehydratase small subunit | 0.032 | Down |
| K09011 | *cimA* | D-citramalate synthase | 0.008 | Down |
| K01652 | *ilvB/G/I* | acetolactate synthase I/II/III large subunit | 0.008 | Down |
| K01653 | *IlvH/N* | acetolactate synthase I/III small subunit | 0.032 | Down |
| K01687 | *ilvD* | dihydroxy-acid dehydratase | 0.032 | Down |
| K00266 | *gltD* | glutamate synthase (NADPH/NADH) small chain | 0.016 | Down |
| K01915 | *glnA* | glutamine synthetase | 0.016 | Down |
| K00930 | *argB* | acetylglutamate kinase | 0.008 | Down |
| K00145 | *argC* | N-acetyl-gamma-glutamyl-phosphate reductase | 0.032 | Down |
| K00818 | *argD* | acetylornithine aminotransferase | 0.008 | Down |
| K01438 | *argE* | acetylornithine deacetylase | 0.008 | Down |
| K01940 | *argG* | argininosuccinate synthase | 0.032 | Down |
| K01755 | *argH* | argininosuccinate lyase | 0.032 | Down |
| K00931 | *proB* | glutamate 5-kinase | 0.032 | Down |
| K00147 | *proA* | glutamate-5-semialdehyde dehydrogenase | 0.008 | Down |
| K00286 | *proC* | pyrroline-5-carboxylate reductase | 0.008 | Down |
| K00163 | *aceE* | pyruvate dehydrogenase E1 component | 0.016 | Up |
| K01834 | *gpmA* | 2,3-bisphosphoglycerate-dependent phosphoglycerate mutase | 0.008 | Up |
| K02446 | *glpX* | fructose-1,6-bisphosphatase II | 0.008 | Up |
| K16305 | *fbpa* | fructose-bisphosphate aldolase | 0.025 | Up |
| K00248 | *bcd* | butyryl-CoA dehydrogenase | 0.016 | Up |
| K01034 | *atoD* | acetate CoA/acetoacetate CoA-transferase alpha subunit | 0.032 | Up |
| K01035 | *atoA* | acetate CoA/acetoacetate CoA-transferase beta subunit | 0.032 | Up |
| K01692 | *paaF* | enoyl-CoA hydratase | 0.021 | Up |
| K01715 | *crt* | enoyl-CoA hydratase | 0.016 | Up |
| K01825 | *fadB* | 3-hydroxyacyl-CoA dehydrogenase / enoyl-CoA hydratase / 3-hydroxybutyryl-CoA | 0.025 | Up |
| K00127 | *fdoI* | formate dehydrogenase subunit gamma | 0.036 | Up |
| K11261 | *fwdE* | formylmethanofuran dehydrogenase subunit E | 0.032 | Up |
| K01694 | *TRP* | tryptophan synthase | 0.016 | Up |
| K12339 | *cysM* | cysteine synthase B | 0.016 | Up |
| K00813 | *aspC* | aspartate aminotransferase | 0.025 | Up |
| K01682 | *acnB* | aconitate hydratase 2 | 0.016 | Up |
| K01948 | *CPS1* | carbamoyl-phosphate synthase | 0.032 | Up |
