# Supplementary material for: Disruption of ruminal homeostasis by malnutrition involved in systemic ruminal microbiota-host interactions in a pregnant sheep model
Source: Microbiome. 2020 Sep 24;8:138. doi: 10.1186/s40168-020-00916-8 (PMC7517653; doi:10.1186/s40168-020-00916-8)

**Additional file 4**

**Supplementary Fig. S3** The PCA and PLS-DA of total genes in the ruminal epithelium of the CON and SFR groups. (a) The PCA score scatter plot; (b) The PLS-DA score scatter plot [predictive ability parameter (Q^2^) (cum)=0.594, goodness-of-fit parameter (R^2^) (Y)=0.980]. PCA, principal components analysis; PLS-DA, partial least squares of discriminant analysis.


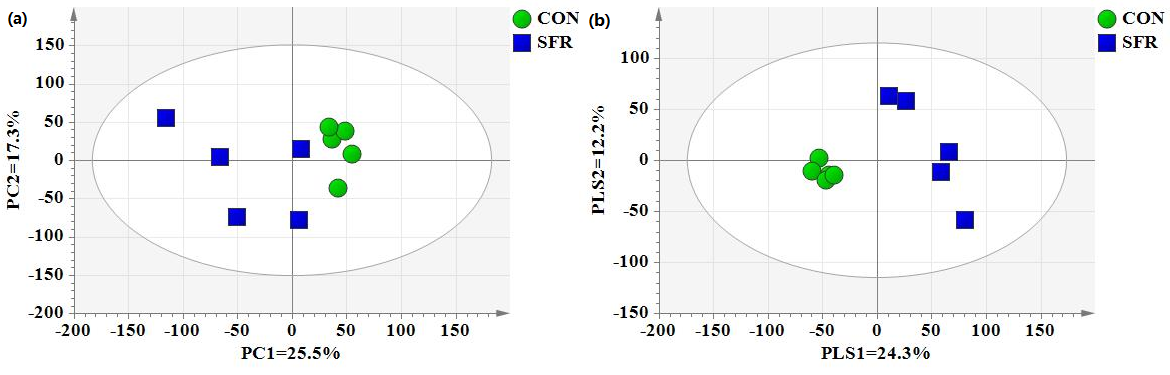

Supplement: Supplementary file 5 — Additional file 4:. Supplementary Fig. S3 The PCA and PLS-DA of total genes in the ruminal epithelium of the CON and SFR groups. (a) The PCA score scatter plot; (b) The PLS-DA score scatter plot [predictive ability parameter (Q2) (cum) = 0.594, goodness-of-fit parameter (R2) (Y) = 0.980]. PCA, principal components analysis; PLS-DA, partial least squares of discriminant analysis. [file 40168_2020_916_MOESM4_ESM.docx]
