# Supplementary material for: Disruption of ruminal homeostasis by malnutrition involved in systemic ruminal microbiota-host interactions in a pregnant sheep model
Source: Microbiome. 2020 Sep 24;8:138. doi: 10.1186/s40168-020-00916-8 (PMC7517653; doi:10.1186/s40168-020-00916-8)
Supplement: Supplementary file 6 — Additional file 5: Supplementary Fig. S4 Validation of RNA-sequencing data using quantitative real-time PCR. Data were represented as the mean with SEM. The difference of gene expression was identified by independent-sample t-test (n = 8 per group), and asterisk indicated the significant difference (P < 0.05). [file 40168_2020_916_MOESM5_ESM.docx]

**Additional file 5**

**Supplementary Fig. S4** Validation of RNA-sequencing data using quantitative real-time PCR. Data were represented as the mean with SEM. The difference of gene expression was identified by independent-sample t-test (n= 8 per group), and asterisk indicated the significant difference (*P* < 0.05).
