# Supplementary material for: Disruption of ruminal homeostasis by malnutrition involved in systemic ruminal microbiota-host interactions in a pregnant sheep model
Source: Microbiome. 2020 Sep 24;8:138. doi: 10.1186/s40168-020-00916-8 (PMC7517653; doi:10.1186/s40168-020-00916-8)
Supplement: Supplementary file 7 — Additional file 6:. Supplementary Table S2 Ingredient composition and nutritional level of the total mixed ration. [file 40168_2020_916_MOESM6_ESM.docx]

**Additional file 6**

**Supplementary Table S2** Ingredient composition and nutritional level of the total mixed ration

| Item | Diet |
| --- | --- |
| Ingredient composition, % DM^1^ | |
| Rye silage | 42.3 |
| Oat hay | 34.6 |
| Maize | 12.0 |
| Soybean meal | 5.8 |
| Barley | 4.2 |
| Premix^2^ | 1.1 |
| Nutrition composition | |
| Digestible energy, MJ/kg DM | 14.78 |
| CP, % of DM | 14.71 |
| Crude fat, % of DM | 2.95 |
| NDF, % of DM | 48.32 |
| ADF, % of DM | 29.09 |
| Crude ash, % of DM | 7.35 |
| Calcium, % of DM | 0.50 |
| Phosphorus, % of DM | 0.37 |

^1^ DM, dry matter basis.

^2^ The premix (per kg) contained: Vitamin A 64.8 mg, Vitamin D_2_ 1.35 mg, Vitamin E 1080 mg, Nicotinic acid 353 mg, Mn 537 mg, Cu 540 mg, Zn 2422 mg, Co 7.2 mg, I 32 mg, Se 18 mg, P 66 g, Ca 130 g, NaHCO_3_ 89 g, NaCl 140~180 g.
