# Supplementary material for: Disruption of ruminal homeostasis by malnutrition involved in systemic ruminal microbiota-host interactions in a pregnant sheep model
Source: Microbiome. 2020 Sep 24;8:138. doi: 10.1186/s40168-020-00916-8 (PMC7517653; doi:10.1186/s40168-020-00916-8)
Supplement: Supplementary file 8 — Additional file 7:. Supplementary Table S3 Gene primers used for quantitative real time-PCR. [file 40168_2020_916_MOESM7_ESM.docx]

**Additional file 7**

**Supplementary Table S3** Gene primers used for quantitative real time-PCR

| Gene symbol | Primer sequence | Accession number | Length (bp) |
| --- | --- | --- | --- |
| *MCM2* | F: GATGTCTATGAGGCTGAGGGG | XM_015102122.1 | 340 |
|  | R: GGAAGTTCTTGAAGCGGTGG |  |  |
| *MCM4* | F: CAGTAGCCGAGGGATGGTGT | XM_012134357.2 | 204 |
|  | R: CCTGTGGTGAGGATGGAGATG |  |  |
| *MCM5* | F: ACGGAGGGGTTGTCTGTATTG | XM_012162077.1 | 124 |
|  | R: TTGAGGGTGGTGGTGATGC |  |  |
| *COL1A1* | F: AGAAGAAGGAAAGCGAGGAGC | XM_015098715.1 | 270 |
|  | R: CCAGTTTTGCCATCAGGACC |  |  |
| *COL1A2* | F: GCTGGCATTCGTGGTTCTC | XM_004007726.3 | 292 |
|  | R: TCCATTCTGGGTGGCTGAG |  |  |
| *COL3A1* | F: TTTTCGCTCTGCTTCATCCC | XM_004004514.3 | 230 |
|  | R: GCAAACTGCACAACATTCTCC |  |  |
| *CPT1* | F: CCTTCCCATTCCGCACTTT | NM_001009414.1 | 171 |
|  | R: CGGTCTCTGTTCTGCCCTCT |  |  |
| *PPARG* | F: ATTTCTGCTCCGCACTACGA | AY137204.1 | 122 |
|  | R: GGGGATACAGGCTCCACTTT |  |  |
| *CDK1* | F: CCAATAATGAAGTGTGGCCAGAAG | NM_174016.2 | 164 |
|  | R: AGAAATTCGTTTGGCAGGATCATAG |  |  |
| *CDK2* | F: CCTAGCTTTCTGCCACTCTCAT | NM_001142509.1 | 153 |
|  | R: TCACCACCTCGTGGGTATAAGT |  |  |
| *CDK4* | F: GACCAAGACCTCAGGACGTATC | NM_001127269.1 | 250 |
|  | R: CACCACTTGTCACCAGAATGTT |  |  |
| *CDK6* | F: GATGGCTCTTACCTCAGTGGTT | XM_012177413.2 | 228 |
|  | R: GGGTAGGGCAACATCTCTAGG |  |  |
| *Cyclin A2* | F: CGGAAGAAGTCAACATCGT | NC_019478.2 | 119 |
|  | R: TAAAGTCTCGGGTAGCAAG |  |  |
| *Cyclin B1* | F: AGCGGATCCAAACCTTTGTAGTG | NM_001045872.1 | 137 |
|  | R: CAATGAGGATGGCTCTCATGTTTC |  |  |
| *Cyclin D1* | F: CCTGCCGTCCATGCGGAA | NC_019478.2 | 403 |
|  | R: GAACTTCACATCTGTGGCAC |  |  |
| *Cyclin E1* | F: TGGCACCGATGTCTCTGTTC | XM_015100542 | 114 |
|  | R: CCACACTGGCTTCTCACAGT |  |  |
| *GAPDH* | F: GGGTCATCATCTCTGCACCT | NM_001034034.2 | 180 |
|  | R: GGTCATAAGTCCCTCCACGA |  |  |
